# Community Structure and Function of Aerobic Methanotrophs in Urban River Sediments: A Case Study of the Jialing River

**DOI:** 10.1111/1758-2229.70167

**Published:** 2025-07-29

**Authors:** Yongliang Mo, Jiuling Huang, Maoli Hu, Lu Lu, Muhammad Shaaban

**Affiliations:** ^1^ Key Laboratory of Nanchong City of Ecological Environment Protection and Pollution Prevention in Jialing River Basin College of Environmental Science and Engineering, China West Normal University Nanchong China; ^2^ Key Laboratory of the Three Gorges Reservoir Region's Eco‐Environment, Ministry of Education College of Environment and Ecology, Chongqing University Chongqing China; ^3^ College of Agriculture, Henan University of Science and Technology Luoyang China

**Keywords:** aerobic methanotrophs, high‐throughput sequencing, Jialing River, microbial community, urban river sediment

## Abstract

Rivers are hotspots of global methane emission and oxidation, yet research on aerobic methanotrophy in urban river sediments remains limited. Here, we collected sediments of the Jialing River and its tributary (Xixi River) from the urban area of Nanchong City (Sichuan Province, China), and used microcosm incubation and high‐throughput sequencing methods to investigate the aerobic methane oxidation rate and the community structure of methanotrophs of these urban river sediments. Our results indicated that these urban river sediments exhibited substantial methane oxidation potential, with the maximum rate observed at the downstream site; high‐throughput sequencing revealed that type I methanotrophs (*Methylobacter*, *Methylomicrobium* and *Crenothrix*) were the key microbial groups responsible for aerobic methane oxidation. The river physico‐chemical properties such as dissolved organic carbon (DOC), total carbon (TC) and C/N ratio correlated significantly with aerobic methane oxidation rates. These findings suggest that urban rivers possess significant methane oxidation potential, which is notably affected by carbon and nitrogen contents. Future research should focus on the metabolic mechanisms of urbanisation on fluvial methanotrophy.

## Graphical abstract

Urban river sediments collected from the downstream sites of Jialing River possess the highest aerobic methane oxidation potential. The potential is affected by river carbon and nitrogen contents. Type I methanotrophs contribute to the activity.

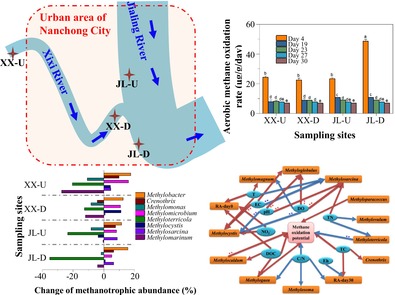

## 1 Introduction

Rivers and streams contribute significantly to global methane (CH_4_) cycling, and thus impact global climate change. Traditionally, it was believed that these well‐ventilated rivers were conducive to CH_4_ oxidation and emitted very little CH_4_. However, recent studies have identified streams and rivers as significant sources of atmospheric methane emissions (Li et al. 2021). It is estimated that during the decade from 2008 to 2017, global methane emissions averaged 576 Tg CH_4_ per year (Saunois et al. 2020), with rivers and streams contributing 27.9 Tg CH_4_ per year (Rocher‐Ros et al. 2023). However, research on methane emission and oxidation in inland water bodies on a global scale has predominantly focused on temperate and cold regions, specifically between 30° N and 75° N, mainly in North America and Europe (Stanley et al. 2016). In addition, wetlands and lakes have been the primary focus of previous studies, with relatively little research conducted on methane cycling in rivers (Bhushan et al. 2024).

Specially, urban river water contains more methane than non‐urban river water due to human activities such as warm sewage discharge (Tang et al. 2021; Zhang et al. 2021). Human activities have multifaceted impacts on river ecosystems, including sediment deposition, nutrient enrichment, pollutant contamination, changes in hydrological characteristics, destruction of riparian vegetation, loss of tree shade, and reduction of large and coarse plant residues (Herrero Ortega et al. 2019; Zhao et al. 2022). River water often contains CH_4_ levels that exceed saturation, which may feed methanotrophs. When methane diffuses from sediment towards water and the atmosphere, it is oxidised by aerobic and anaerobic methane oxidising bacteria (Conrad 2009; Knittel and Boetius 2009).

Aerobic methanotrophs contribute significantly to methane consumption in flowing shallow habitats where O_2_ is available, including the near‐shore river sediments. These bacteria can be categorised into the phyla Proteobacteria (type I γ‐Proteobacteria and type II α‐Proteobacteria) and Verrucomicrobia based on their cell structure and function (Hanson and Hanson 1996; Knief 2015). Additionally, type I and II methanotrophs possess different growth strategies. Type I methanotrophs prefer high methane concentration, while type II methanotrophs can utilise low atmospheric CH_4_ due to their high‐affinity methane oxidation enzymes (Ho et al. 2013; Cai et al. 2016). In some UK river sediments, *Methylobacter* and *Methylomonas* that belong to type I were reported to be active microbes after methane oxidation (Sherry et al. 2016). However, this was challenged by Gou et al. (2023); they showed that type II methanotrophs (*Methylocystis*) dominated aerobic methanotrophs of the urban river sediments of the Yangtze River (China). The anaerobic methanotrophic archaea (ANME), however, were confined to function in deep anoxic sediment layers (Guerrero‐Cruz et al. 2021). Shen et al. (2019) reported anaerobic methane oxidation potentials in England river sediments; they were significantly lower than aerobic methane oxidation potentials of Wuxijiang (China) river sediments (He et al. 2024). Nevertheless, it remains obscure what the magnitude of aerobic methane oxidation potentials and their corresponding functional guilds, as well as the impact factors in urban rivers. Furthermore, previous research indicates that methane cycling in rivers is regulated by multiple factors, including CH_4_, dissolved oxygen (DO), temperature, pH, ammonium (NH_4_^+^), phosphorus (P), CO_2_, and nitrate (NO_3_^−^) (Campeau and Giorgio 2014; Gou et al. 2023; Patel et al. 2024). For instance, nitrite significantly correlated with methanotrophs in the urban river of Chongqing (China) (Ouyang et al. 2023); Robison et al. (2021) found the rates of methane ebullition increased with temperature. They explained this phenomenon by Q10 values of the methane ebullition process. Yet we cannot discriminate the effects of temperature on methanotrophs out of methanogens, as they often work simultaneously to control overall methane exchange in rivers. Additionally, the variations of sedimentary physicochemical properties (e.g., dissolved oxygen, CH_4_ concentration, carbon, nitrogen, Eh, and temperature etc.) would further complicate aerobic methanotrophy in urban river sediments.

Based on the importance and information gaps in urban river methanotrophy, this study selected the Jialing River, a significant tributary of the upper reaches of the Changjiang River in China, as the research object. Sediments from the urban river section in Nanchong City were collected, and microcosm incubation and high‐throughput sequencing were conducted to investigate the methane oxidation rate, functional microorganisms, and their influencing factors in urban river sediments. We hypothesised that: (1) Urban river sediments possess methane oxidation potentials; (2) Type I methanotrophs that prefer high methane concentrations are responsible for methanotrophy; (3) Sediment properties are expected to mediate the methane oxidation of these samples. This research possesses significance in understanding the methane oxidation mechanisms in urban river ecosystems.

## 2 Materials and Methods

### 2.1 Sampling

**FIGURE 1:**
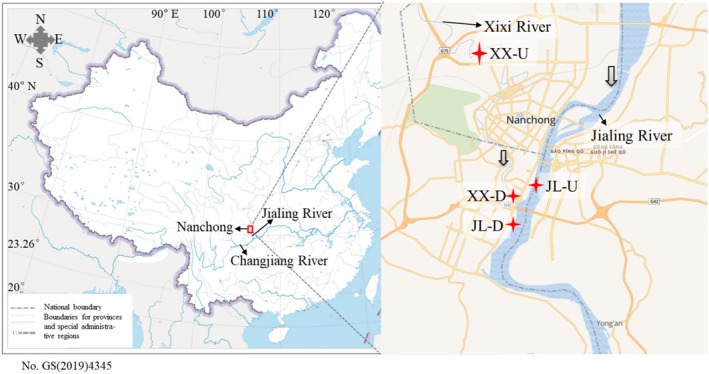
Map of sampling sites of urban river sediments. We collected four sediments from Jialing River and its tributary Xixi River: (1) the sediments sample XX‐U is collected from the Xixi River before it flows into the urban area, located in Huafeng Town, Shunqing District; (2) The XX‐D sampling point is situated before the confluence of the Xixi River into the Jialing River, under the Huanzi River Bridge in Jialing District; (3) The JL‐U sample is collected from the Jialing River before the confluence with the Xixi River, near Nanmenba Wetland Park in Shunqing District; (4) The JL‐D sample is collected from the Jialing River after the confluence of the Xixi River, located next to Wuyun Square in Jialing District, Nanchong. The sampling sites are marked with red cross in the right panel. The grey arrow in the right panel indicates the flow direction of river.

River sediments were collected in June 2021 along the Nanchong urban section of the Jialing River and its tributary, the Xixi River. The Jialing River is an important upstream tributary of the Changjiang River in China (Figure 1). Nanchong City has the second largest population in Sichuan Province. Thus, we selected the following four sampling sites here as typical urban river sediments: XX‐U, XX‐D, JL‐U and JL‐D (see the locations of these sites in Figure 1).

For the convenience of sampling, the sampling sites were in the river channel at a distance of 1~2 m off the bank. The water depths of these sites were 1~3 m. Triplicate 0–10 cm surface sediment samples were collected with a Petersen grab bucket sediment collector with a rope (Schloesser and Thomas 2002). Water samples were also collected into plastic bottles for further physicochemical properties analysis, while sediments were transferred into plastic bags using a plastic sampling shovel from the sediment collector. The numbers on the bags and bottles were checked and recorded. Then we discharged air from the bag after sediment collection. These water and sediment samples were transferred into a thermal‐insulating foam incubator with ice packs and were promptly transported back to the laboratory.

After picking out external matters from sediment samples and mixing triplicated sediment or water samples together for each site, a portion of the sediment samples were used for microbial DNA extraction and frozen at −20°C, while the remaining samples were stored in a refrigerator at 4°C for subsequent analysis.

### 2.2 Analysis of Physical and Chemical Properties of Samples

On‐site measurement of water quality indicators was conducted using a multifunctional AZ86031 water quality meter (AZ Instrument Inc., Taichung, China), which included parameters such as pH, dissolved oxygen (DO), conductivity (EC), and temperature (T). The redox potential (Eh) was measured by an AZ8552 redox potential tester (AZ Instrument Inc., Taichung, China). Nitrate (NO_3_^−^), nitrite (NO_2_^−^) and sulphate (SO_4_^2−^) contents were measured after 0.45 μm filtration using an ICS‐2100 ion chromatograph (Thermo Fisher Scientific, Waltham, MA, USA). Dissolved organic carbon (DOC) was measured using a TOC & Water Analysis instrument (Elementar Analysensysteme GmbH, Langenselbold, Germany). Total carbon (TC) and total nitrogen (TN) were analysed by a carbon and nitrogen element analyser (Elementar Analysensysteme GmbH, Langenselbold, Germany). The physicochemical properties of river water are shown in Table 1.

**TABLE 1:** Physico‐chemical properties of urban river water and sediment samples in this study.

Properties	XX‐U	XX‐D	JL‐U	JL‐D
pH	8.1 ± 0.1 bc	7.9 ± 0.00 c	8.4 ± 0.1 a	8.1 ± 0.00 b
EC, μS/cm	508.7 ± 0.5 b	536.0 ± 7.1 a	369.7 ± 1.2 d	426.7 ± 2.1 c
T, °C	23.8 ± 0.1 a	23.2 ± 0.1 b	21.6 ± 0.00 d	21.9 ± 0.1 c
Eh, mV	86.7 ± 0.9 c	126.7 ± 0.9 a	113.3 ± 1.2 b	127.3 ± 1.7 a
DO, mg/L	6.7 ± 0.2 c	5.6 ± 0.2 d	10.1 ± 0.1 a	8.4 ± 0.4 b
DOC, mg/L	16.93 ± 0.02 b	16.40 ± 0.05 c	15.62 ± 0.01 d	20.63 ± 0.03 a
TC, g/kg	14.16 ± 0.27 b	14.45 ± 0.18 b	17.18 ± 0.46 a	18.00 ± 0.79 a
NO_3_^−^, mg/L	2.03 ± 0.46 b	1.96 ± 0.73 b	2.40 ± 0.14 ab	3.23 ± 0.19 a
NO_2_^−^, mg/L	0.044 ± 0.02 b	0.063 ± 0.03 b	0.0003 ± 0.00 b	0.043 ± 0.01 a
TN, g/kg	0.75 ± 0.02 a	0.48 ± 0.02 c	0.58 ± 0.02 b	0.75 ± 0.06 a
SO_4_^2−^, mg/L	17.17 ± 3.32 b	17.29 ± 5.47 b	21.30 ± 1.08 ab	27.56 ± 2.07 a

*Note:* XX‐U and XX‐D are respectively, the upstream and downstream sites of the Xixi River; JL‐U and JL‐D are respectively, the upstream and downstream sites of the Jialing River. The means followed by different letters within the same line indicates statistical significance at the level of *p* < 0.05 (LSD).

Abbreviations: DO, dissolved oxygen; DOC, dissolved organic carbon; EC, electron conductivity; Eh, redox potential; NO_2_^−^, nitrite; NO_3_^−^, nitrate; SO_4_^2−^, sulphate; T, temperature; TC, total carbon; TN, total nitrogen.

### 2.3 Microcosm Incubation

The microcosm incubation method was used to determine methane oxidation rate (Mo et al. 2020). Fresh sediment (equivalent to 6.00 g dry weight) was placed into 120 mL bottles, sealed with rubber stoppers, and pre‐incubated in the dark at 28°C for 24 h to activate microbial activity. After pre‐incubation, 13.0 mL of air was removed from each bottle and replaced with an equal volume of methane gas (CH_4_, purity greater than 99.9%) to achieve a 10% (V/V) CH_4_ concentration. We set up this concentration to simulate high methane conditions in the sediment (Rulik et al. 2013; Mach et al. 2015). Each treatment was repeated three times and incubated at 28°C ± 0.5°C in a SPX 250B thermostat incubator (Hongnuo Inc., Tianjin, China), as most methanotrophs grow optimally under this temperature (Dave et al. 2025).

At the start of incubation, methane gas was injected and mixed thoroughly. Zero‐time gas samples, representing the initial CH_4_ concentration, were collected immediately. The CH_4_ concentration in the headspace gas of the culture bottle was then dynamically measured over time at day 4, 19, 23, 27 and 30. We observed steady methane concentration after 27–30 days. Soil samples were collected destructively for further analysis at day 30. The methane concentration in the culture bottle was measured using a SHA‐12 gas chromatograph (Yidian Inc., Shanghai, China). Upon completion of the incubation, the soil samples were stored at −80°C for DNA extraction and subsequent high‐throughput sequencing.

Methane oxidation rate (*R*_methane_) was calculated using Formula (1):

(1) R=methaneCi−Ce×V×ρmethanem×t

where *R*_methane_ is methane oxidation rate, μg CH_4_ g^−1^ (dry weight soil) day^−1^; *C*_i_ and *C*_e_ were respectively, the methane concentration of the initial and end of the incubation, μL L^−1^; *V* is the volume of bottle headspace, L; *ρ*_methane_ is the density of methane in standard conditions (1.01 × 10^5^ Pa, 273.15 K), 0.72 g L^−1^; *m* is the dry mass of sediment, g; *t* is the incubation days.

### 2.4 Microbial DNA Extraction and High Throughput Sequencing

#### 2.4.1 DNA Extraction

Microbial DNA from the sediment was extracted following the instructions of the PowerSoil DNA Isolation Kit (Mo‐Bio Laboratories Inc., Carlsbad, CA, USA). The quality and concentration of DNA were checked using 1% agarose gel electrophoresis and spectrophotometric methods (NanoDrop 2000, NanoDrop Technologies, Wilmington, DE, USA). DNA samples were stored at −80°C for further use.

#### 2.4.2 PCR Amplification

The primer pair 515F/907R was used to amplify the V4–V5 regions of sedimentary microbial 16S rRNA genes (Christner et al. 2001). An 8‐bp barcode was added at the 5′ end of the primer to identify samples. The 25 μL PCR reaction mixture consisted of 12.5 μL KAPA 2G Robust Hot Start Ready Mix (Kapa Biosystems, Wilmington, MA, USA), 1 μL forward primer (5 μM), 1 μL reverse primer (5 μM), 5 μL DNA (total DNA 30 ng), and 5.5 μL ddH_2_O. The primers were synthesised by Takara Bio Inc. (Kusatsu, Japan). The temperature programme was as follows: 95°C for 5 min; followed by 28 cycles of 95°C for 45 s, 55°C for 50 s, and 72°C for 45 s; and a final extension at 72°C for 10 min (Christner et al. 2001). The size of the PCR products was verified by agarose gel electrophoresis method and purified using Agencourt AMPure XP kit (Beckman Coulter Life Sciences, Indianapolis, IN, USA).

#### 2.4.3 NovaSeq Sequencing

PCR products were used to construct a library and subjected to paired‐end sequencing on the Illumina NovaSeq PE250 platform (Allwegene Technology, Beijing China). Raw sequence data were uploaded to the NCBI‐SRA database (SSR 19554148—SRR 19554099, BioProject accession: PRJNA 846137). The website for this database is available at https://www.ncbi.nlm.nih.gov/sra.

#### 2.4.4 Data Processing

Sequence data was processed using QIIME2 (version 2021.4) (Bolyen et al. 2019). The online tutorials are available at the website: https://docs.qiime2.org/2024.10/ tutorials/index.html. Briefly, the demultiplexed samples were merged, quality‐filtered, chimera checked, and denoised by DATA2 following the official tutorials (Callahan et al. 2016). Samples were rarefied based on the least reads number of 53 595. ASV taxonomy annotation was performed by sklearn classifier against the SILVA_138_1 database (Quast et al. 2012). Aerobic methanotrophs were manually identified based on the references (Hanson and Hanson 1996; Knief 2015; Dedysh and Knief 2018; Farhan Ul Haque et al. 2020; Guerrero‐Cruz et al. 2021; Yao et al. 2023; Ahmadi and Lackner 2024).

One‐way ANOVA (analysis of variance) followed by post hoc least significant difference (LSD) tests was performed to compare the statistical differences of physico‐chemical properties and relative abundances between sampling sites using SPSS software (version 24.0, IBM Corp., Armonk, NY, USA). We also performed Spearman correlation analysis to analyse the relationship between methanotrophs and environmental factors using SPSS 24.0. We used Origin 2019 for graphic drawing (OriginLab Corporation, Northampton, MA, USA).

## 3 Results

### 3.1 Physico‐Chemical Properties

As showing in Table 1, the Jialing River samples (JL‐U and JL‐D) possessed higher pH, dissolved oxygen (DO), redox potential (Eh), carbon, nitrogen, and sulphate contents than that of the Xixi River (XX‐U and XX‐D). On the contrary, the Xixi River samples had significantly higher temperature and electrical conductivity (EC) than the Jialing River (Table 1). Furthermore, DO in the upper sites of both rivers was significantly higher than in the downstream sites. However, EC, Eh and DOC exerted opposite paradigms between the upstream and downstream samples from both rivers (Table 1). There was no significant difference in SO_4_^2−^, NO_3_^−^ and total carbon (TC) values between the upstream and downstream sites of both rivers (Table 1).

### 3.2 Aerobic Methane Oxidation

**FIGURE 2:**
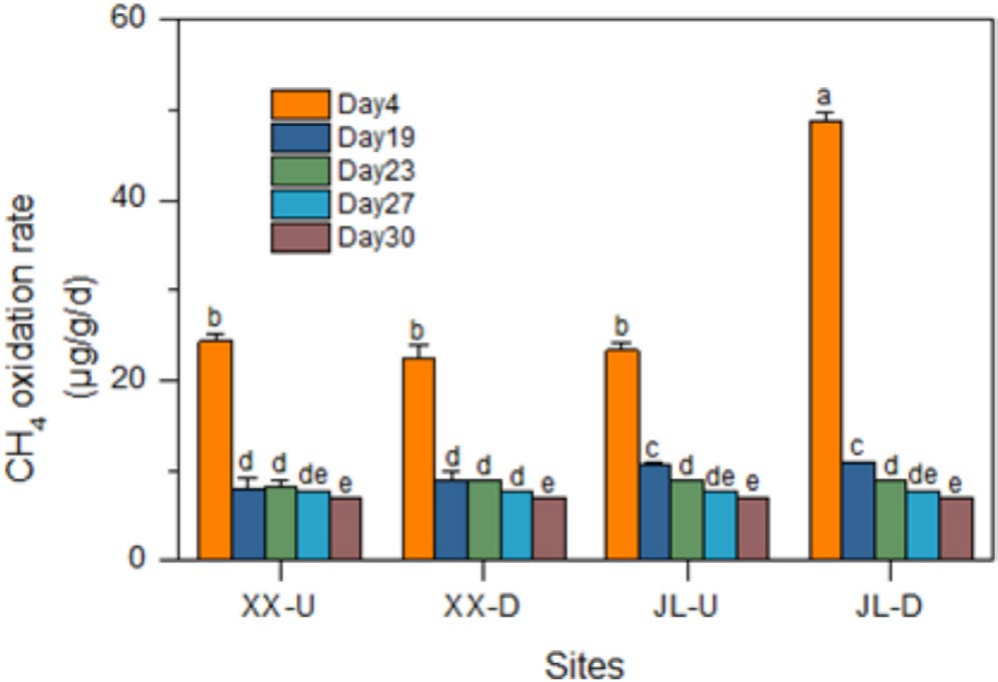
Variations in methane oxidation rate with incubation time in Jialing River sediment. Different letters above the bar chart indicate significant differences in numerical values (LSD, *p* < 0.05). See the sediment codes in Figure 1.

During the first 4 days of incubation, the methane oxidation rate of the downstream sample (JL‐D) was significantly higher than that of the other upstream samples, with no significant differences observed among the three upstream samples (Figure 2). Interestingly, we noted significantly higher rates in the Jialing River sediments than that of the Xixi River after 19 days’ incubation; after 23, 27 and 30 days of incubation, there was no significant difference between sites despite a significant downward trend in rate with incubation days (Figure 2).

Within the overall 30 days of incubation, the JL‐D site exhibited the highest methane oxidation rate of 48.7 μg/g/d. The average methane oxidation rate across all sites was 6.89 μg/g/d (Figure 2).

### 3.3 Aerobic Methanotrophic Community

**FIGURE 3:**
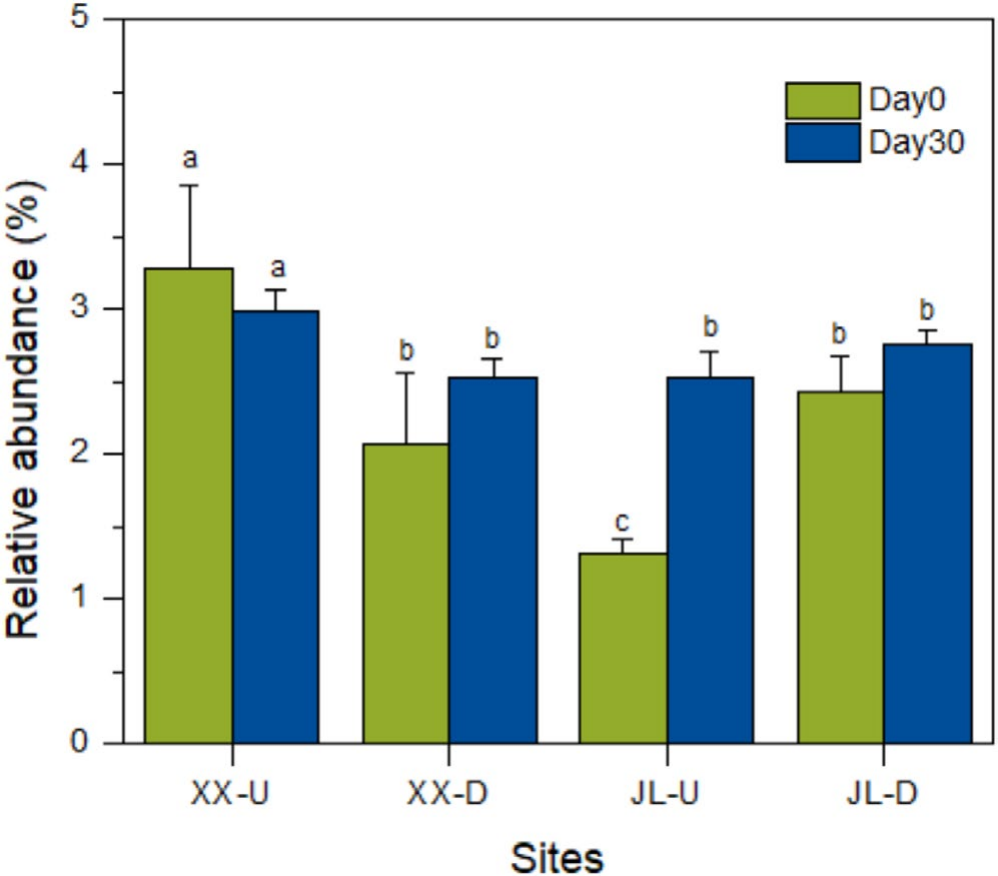
Relative abundances of total aerobic methanotrophs in Jialing river sediment. Different letters above the bar chart indicate significant differences in numerical values (LSD, *p* < 0.05). See the sediment codes in Figure 1.

The relative abundance of total methane oxidising bacteria at three out of four sampling points showed an increasing trend after 30 days of methane oxidation, with a significant growth observed only in the JL‐U sediment. Conversely, a decrease was observed in the XX‐U sample (Figure 3).

**FIGURE 4:**
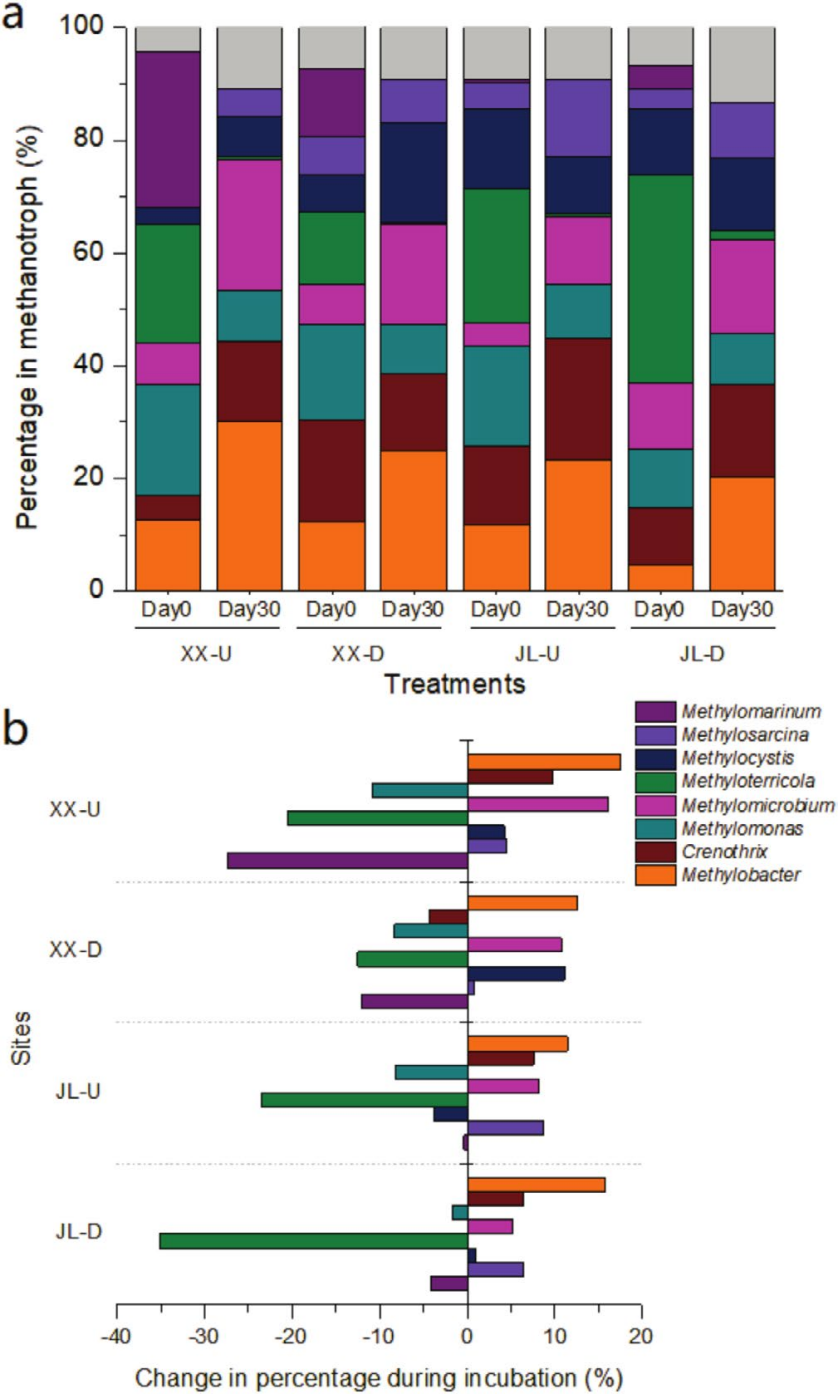
Percent compositions (a) and variations (b) of aerobic methanotrophs after 30 days of methane oxidation in Xixi and Jialing River sediments. The grey colour in panel (a) represents rare taxa with the percent abundance less than5%. See the sediment codes in Figure 1.

The percent compositions of aerobic methane oxidising bacteria in sediments at day 0 reflected their initial environmental niches. The Xixi River samples (XX‐U and XX‐D) were initially distributed more evenly in the relative abundances than the Jialing River samples (JL‐U and JL‐D) (Figure 4a). Specifically, XX‐U samples were primarily composed of *Methylomarinum* (27.4%), *Methyloterricola* (21.1%) and *Methylomonas* (19.8%). In the XX‐D sample, *Crenothrix* (17.9%) and *Methylomonas* (17.0%) were more prevalent than other methanotrophs. The JL‐U community was predominantly composed of *Methyloterricola* (23.8%) and *Methylomonas* (17.8%). Similarly, *Methyloterricola* accounts for 36.8% of the JL‐D community (Figure 4a).

After 30 days of methane consumption, the dominant aerobic methane oxidising bacteria in the sediments reflected the competition winners in methane oxidation, potentially playing a more crucial role. We observed that the percentages of *Methylobacter*, *Methylomicrobium* and *Methylosarcina* increased in all samples (Figure 4b). Notably, the genus *Methylobacter* increased by 17.5%, 12.5%, 11.4% and 15.7% in the XX‐U, XX‐D, JL‐U and JL‐D samples, respectively. These methanotrophs were probably involved in methane oxidation and were functionally important.

### 3.4 Spearman Correlations Between Aerobic Methanotrophy and Physico‐Chemical Properties

We observed statistically significant correlations between methane oxidation rate and the contents of total carbon (TC), dissolved organic carbon (DOC) and carbon to nitrogen ratio (C/N), with the correlation coefficients of 0.72 (*p* < 0.01), 0.70 (*p* < 0.05) and −0.64 (*p* < 0.05), respectively (Table 2). Surprisingly, the relative abundance of total methanotrophs (RA) at day 0 and day 30 showed significant correlations with temperature (T), DOC, NO_2_^−^, TC, Eh and C/N ratio, not significant with the methane oxidation potential (Table 2).

**TABLE 2:** Spearman correlations between aerobic methane oxidation potential, relative abundance of total methanotroph (RA) and physico‐chemical properties of urban river sediments and water.

Parameters	MOP			RA‐day0			RA‐day30		
	*r*	*P*	*N*	*r*	*P*	*N*	*r*	*p*	*N*
pH	0.17	0.602	12	−0.40	0.199	12	0.45	0.145	12
T	0.07	0.836	12	**0.74**	**0.006**	**12**	0.03	0.922	12
EC	−0.20	0.527	12	0.45	0.147	12	−0.36	0.254	12
Eh	0.10	0.753	12	−0.13	0.687	12	**−0.62**	**0.031**	**12**
DO	0.18	0.585	12	−0.43	0.165	12	0.43	0.165	12
TC	**0.72**	**0.008**	**12**	0.44	0.152	12	**0.59**	**0.045**	**12**
DOC	**0.70**	**0.011**	**12**	**0.68**	**0.015**	**12**	0.04	0.897	12
TN	0.41	0.191	12	−0.36	0.255	12	−0.16	0.618	12
C/N	**−0.64**	**0.026**	**12**	−0.50	0.101	12	**−0.62**	**0.033**	**12**
NO_3_^−^	0.48	0.118	12	0.23	0.471	12	−0.14	0.665	12
NO_2_^−^	−0.16	0.617	12	**0.66**	**0.019**	**12**	−0.38	0.230	12
SO_4_^2−^	0.45	0.145	12	0.06	0.863	12	−0.20	0.527	12
RA‐day0	0.11	0.74	12						
RA‐day30	−0.13	0.69	12						

*Note:* The orange colour indicates significant and positive correlation (*p* < 0.05); the light orange colour indicates positive correlation (not statistical significant); the blue colour indicates significant and negative correlation (*p* < 0.05); the light blue colour indicates negative correlation (not statistical significant). The bold value indicates significant correlation (*p* < 0.05).

Abbreviations: C/N, total carbon to total nitrogen ratio; DO, dissolved oxygen; DOC, dissolved organic carbon; EC, electron‐conductivity; Eh, redox potential; MOP, aerobic methane oxidation potential; NO_2_^−^, nitrite; NO_3_^−^, nitrate; RA‐day0 and RA‐day30, the relative abundances of total methanotrophs at day 0 and day 30; SO_4_^2−^, sulphate; T, temperature; TC, total carbon; TN, total nitrogen.

Therefore, we further checked the relationship between methanotrophic taxa change (relative abundances of genera), physico‐chemical properties, and methane oxidation potentials of sediments. We found significant and positive correlations between methane oxidation potentials and *Methylocaldum* (*r* = 0.73, *p* < 0.01), *Methylogaea* (*r* = 0.62, *p* < 0.05) and *Methyloparacoccus* (*r* = 0.60, *p* < 0.05) (Table 3). However, the correlation between methane oxidation potential and *Methyloterricola* was significant and negative (*r* = −0.63, *p* < 0.05) (Table 3). As expected, river properties such as DO, DOC, TC, TN, C/N, pH, EC, T, etc. presented significant correlations with one or more methanotrophic genera (Table 3). Among them, DO was significantly correlated with *Methylosarcina* (*r* = 0.81, *p* < 0.01), *Methyloglobulus* (*r* = 0.73 *p* < 0.01), *Methylocystis* (*r* = −0.79, *p* < 0.01) and *Methylomagnum* (*r* = −0.63, *p* < 0.05) (Table 3). Carbon and nitrogen possessed close and complicated relationships with methanotrophs. River carbon was generally positively related to methanotrophs (e.g., DOC vs. *Methylogaea* (*r* = 0.69, *p* < 0.05), DOC vs. *Methylocaldum* (*r* = 0.62, *p* < 0.05), TC vs. *Crenothrix* (*r* = 0.59, *p* < 0.05)). However, TN had both positive and negative correlations with methanotrophs. Similarly, the correlations between pH, T and methanotrophs were either positive or negative depending on the specific taxa (Table 3).

**TABLE 3:** Spearman correlation analysis between the changes in relative abundances of methanotrophic genera, aerobic methane oxidation potential and physico‐chemical properties of urban river sediments and water.

Genera	MOP	DO	TC	DOC	TN	C/N	NO_3_^−^	NO_2_^−^	SO_4_^2−^	pH	EC	T	Eh
*Crenothrix*	0.22	0.39	0.59*	0.14	0.01	−0.57	−0.08	−0.14	−0.06	0.35	−0.35	0.13	−0.50
*Methylobacter*	0.13	−0.05	0.25	0.22	−0.09	−0.24	0.27	0.38	0.15	−0.06	0.07	0.38	−0.08
*Methylocaldum*	0.73**	0.00	0.34	0.62*	0.43	−0.18	0.37	0.16	0.37	−0.05	0.00	0.02	0.40
*Methylococcus*	0.28	−0.26	−0.08	0.36	−0.34	−0.01	−0.29	−0.01	−0.29	−0.19	0.18	0.23	−0.10
*Methylocystis*	−0.28	−0.79**	−0.24	0.11	−0.51	0.18	−0.13	0.68*	−0.25	−0.72**	0.86**	0.59*	0.27
*Methylogaea*	0.62*	−0.23	0.53	0.69*	−0.26	−0.64*	−0.06	0.19	−0.18	−0.18	0.17	0.48	−0.21
*Methyloglobulus*	−0.17	0.73**	0.08	−0.35	0.20	−0.06	0.06	−0.42	0.11	0.71**	−0.70*	−0.39	−0.28
*Methylomagnum*	−0.04	−0.63*	−0.06	0.42	−0.52	−0.05	−0.33	0.19	−0.37	−0.70*	0.56	0.60*	−0.16
*Methylomarinum*	−0.05	0.07	−0.43	−0.31	0.20	0.51	−0.18	−0.51	−0.03	0.08	−0.11	−0.56	0.23
*Methylomicrobium*	−0.03	−0.32	0.07	0.18	−0.18	−0.20	0.00	0.35	−0.16	−0.25	0.25	0.40	−0.11
*Methylomonas*	0.26	0.07	0.15	0.37	0.51	−0.04	0.29	0.00	0.34	−0.06	−0.03	−0.24	0.56
*Methyloparacoccus*	0.60*	0.21	0.48	0.47	0.55	−0.41	0.34	0.03	0.38	0.08	−0.26	−0.05	0.16
*Methylosarcina*	0.08	0.81**	0.03	−0.32	0.65*	0.09	0.29	−0.52	0.46	0.71**	−0.85**	−0.69*	−0.06
*Methylosinus*	−0.23	0.52	0.24	−0.12	0.22	−0.25	0.31	−0.06	0.27	0.45	−0.48	−0.14	−0.19
*Methylosoma*	0.37	0.32	0.45	0.23	0.04	−0.61*	−0.01	−0.13	0.01	0.22	−0.46	0.18	−0.48
*Methyloterricola*	−0.71**	−0.45	−0.48	−0.48	−0.69*	0.39	−0.50	0.05	−0.55	−0.29	0.52	0.18	−0.21
*Methyloversatilis*	0.33	−0.13	0.04	0.55	0.27	0.04	0.22	0.02	0.24	−0.19	0.15	−0.15	0.56
*Methylovulum*	0.04	−0.28	−0.16	−0.09	−0.59*	−0.07	−0.39	0.14	−0.36	−0.19	0.13	0.40	−0.53

*Note:* The blue colour indicates negative correlation, while the red colourindicates positive correlation; The cell filled with darker colour represents stronger correlation.

Abbreviations: C/N, total carbon to total nitrogen ratio; DO, dissolved oxygen; DOC, dissolved organic carbon; EC, electron‐conductivity; Eh, redox potential; MOP, methane oxidation potential; NO_2_^−^, nitrite; NO_3_^−^, nitrate; SO_4_^2−^, sulphate; T, temperature; TC, total carbon; TN, total nitrogen.

* *P* < 0.05; *N* = 12.

** *P* < 0.01.

## 4 Discussion

Our microcosm incubation of the Jialing and Xixi River sediments showed that the urban river sediments possessed significant aerobic methane oxidation potentials, with higher potentials at the downstream sites. The average rate of 6.89 μg/g/d in our study is comparable with the oxidation rates of the urban sediments collected from Wuxijiang River (China) (0.39–7.89 μg/g/d) (He et al. 2024). However, we recognised that the decreasing trend of methane oxidation rates with incubation time is due to limited methane supply since we only inject methane at the start of the experiment (day‐0). This differs from the in situ environment where methane is diffused from deeper sediment layers to form equilibrium of methane concentration. The higher oxidation rates at downstream sites might be partially caused by the change of river physicochemical properties when the river flows through the urban area of Nanchong City. Different concentrations of pollutants or nutrients such as organic carbon, nitrogen, and phosphorus are involved in river water and sediment at the urban, forest, grassland, and farmland segment sites (Tang et al. 2021). These pollutants or nutrients disturb river physicochemical properties and raise methane concentration in river water and sediments (Stanley et al. 2016; Begum et al. 2021). For instance, a higher content of bioavailable carbon could enhance methane production (Conrad 2007; Zhang et al. 2024). Consequently, urban rivers have been frequently reported as hotspots of methane emission (Li et al. 2021; Tang et al. 2021; Zhao et al. 2022, 2025; Rocher‐Ros et al. 2023).

Moreover, a significant and positive correlation between methane concentration and aerobic methane oxidation rate has been observed by Patel et al. (2024). Methane sustains the activities of the methanotrophic community. Thus, the positive correlations between TC, DOC contents and methane oxidation potential seem to be indirect in our case. This significant correlation is seldom reported in fluvial systems. However, DOC is positively correlated with methane oxidation rates in semiarid soils (USA) during the wet season (Sullivan et al. 2013). The selective organic carbon oxidation by high‐ and low‐affinity methanotrophs may explain this positive correlation. In addition, facultative methanotrophs might contribute to the correlation. Although most methanotrophs utilise methane as a sole carbon and energy source (Hanson and Hanson 1996), there exist facultative methanotrophs that can metabolise dissolved organic carbon such as methanol (methylotrophs) (Chistoserdova and Kalyuzhnaya 2018; Farhan Ul Haque et al. 2020). Furthermore, DOC may affect methane metabolites equilibrium by enabling the growth of carbon metabolising microbes in the riverine ecosystem (Reis et al. 2022). DOC in rivers is highly bioavailable (Liu and Wang 2022), which serves as the substrate for most microorganisms, including methanogens in the Changjiang River (China) (Su et al. 2024); DOC affects methane metabolisms in the discharging, impounding, drying, and flooding periods of the Three‐Gorges Reservoir (China) (Zhang et al. 2024).

In line with the previous study by Long et al. (2017), we showed that the C/N ratio was negatively correlated to methane oxidation activity. Under high C/N conditions, nitrogen deficiency would inhibit methane production and oxidation (Bodelier and Laanbroek 2004; Chen et al. 2024). The important role of nitrate and ammonium has been addressed; they support the growth of methanotrophs in sediment and soil systems, thus affecting methane oxidation rate (Liu et al. 2015; López et al. 2019). However, we did not observe significant effects of nitrate or total nitrogen on methane oxidation rates. The insignificant correlation does not mean that nitrogen (or pH, temperature and so forth) has no effects on methane oxidation due to our sample size limitation.

**FIGURE 5:**
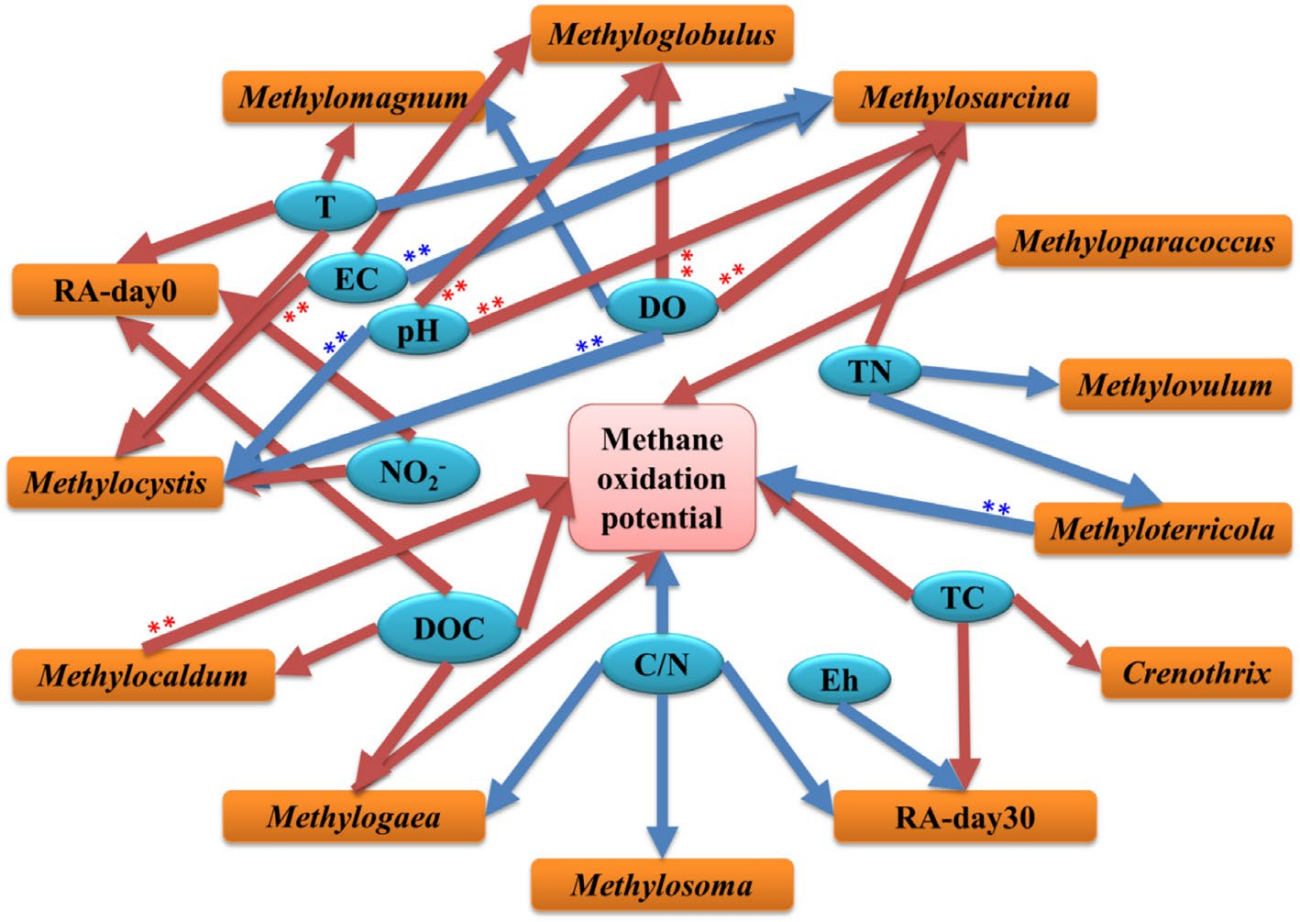
Diagram for the Spearman correlations between methane oxidation potential, physico‐chemical property and change in relative abundance of methanotrophs in urban river water and sediment. Direction of arrow indicates causal link. We hypothesised that physico‐chemical properties could affect methane oxidation potential and methanotroph. And methane oxidation potential was impacted by methanotroph. The red arrow indicates significant and positive correlations (*p* < 0.05). The blue arrow indicates significant and negative correlations (*p* < 0.05). The width of arrow is in proportion to the coefficient (*r*‐value). **, *p* < 0.01.

In addition to the aforementioned physical and chemical factors that affect aerobic methane oxidation rate, microorganisms can also influence this process (Figure 5). Different methanotrophic genera have distinct habitat preferences, leading to variations in their relative abundances and activities under different conditions (Ho et al. 2017). Members of type I methanotrophs have been hypothesised to dominate the methanotrophic communities during high concentration methane oxidation. We showed that *Methylobacter*, *Crenothrix*, and *Methylomicrobium* lead the dominance. *Methylobacter* can combine methane oxidation with aerobic respiration, thereby maintaining energy production in micro‐aerobic or hypoxic environments (Martinez‐Cruz et al. 2017; Smith et al. 2018). Both *Methylobacter* and *Methylomicrobium* are identified to be the functional microorganisms in the aerobic methane oxidation process in rivers of the Yellow River Delta (Hao et al. 2020). Kits et al. (2015) found that *Methylomicrobium* can use NO_3_^−^ as an oxidant for respiration under hypoxic conditions. Oswald et al. (2017) show that *Crenothrix* plays a crucial role in the methane oxidation of two oxygen‐deficient lakes (Lake Rotsee and Lake Zug, Switzerland).

Although our study found that the aerobic methane oxidation rate of urban river sediments in the Nanchong section of the Jialing River is influenced by various physical and chemical properties, and identified some functional aerobic methane oxidising bacteria, the intricate relationships and interactions between microorganisms and their physical and chemical properties warrant further investigation and research. Moreover, due to the limitations of our study (e.g., small sample size (*N* = 12) and limited physicochemical properties analysis, etc.), these correlations need to be further testified. Additionally, under global warming context, it is crucial to illustrate the metabolic mechanisms of methanotrophs to anthropogenic activities during urbanisation.

## 5 Conclusions

Our findings demonstrated significant aerobic methane oxidation potentials in the urban river sediments (the Nanchong section of the Jialing River the Xixi River); specifically, the highest oxidation rate was observed in the downstream samples. The relative abundances of type I methanotrophic genera such as *Methylobacter*, *Methylomicrobium* and *Crenothrix* increased significantly after 30 days' of methane oxidation. They were functionally important during methane oxidation in these urban river sediments. The river physico‐chemical properties, e.g., DOC, TC and C/N ratio showed significant correlations with the rate of aerobic methane oxidation and methanotrophs.

## Author Contributions

**Yongliang Mo:** writing – review and editing, validation, formal analysis, data curation, conceptualization, resources, funding acquisition, project administration. **Jiuling Huang:** writing – original draft, software, methodology, visualization. **Maoli Hu:** writing – original draft, investigation, software, methodology, visualization. **Lu Lu:** writing – review and editing. **Muhammad Shaaban:** writing – review and editing.

## Conflicts of Interest

The authors declare no conflicts of interest.

## Data Availability

The data that support the findings of this study are available on request from the corresponding author. The data are not publicly available due to privacy or ethical restrictions.
